# The Role of Osteoimmunology in Periodontal Disease

**DOI:** 10.1155/2013/639368

**Published:** 2013-09-17

**Authors:** Rayyan A. Kayal

**Affiliations:** Department of Oral Basic and Clinical Science, King Abdulaziz University Faculty of Dentistry, P.O. Box 3738, Jeddah 21481, Saudi Arabia

## Abstract

Periodontal disease is a pathological condition that involves inflammation of the tooth supporting structures. It occurs in response to the presence of bacterial plaque on the tooth structure. The host defense system, including innate and adaptive immunity, is responsible for combating the pathologic bacteria invading the periodontal tissue. Failure to eradicate the invading pathogens will result in a continuous state of inflammation where inflammatory cells such as lymphocytes, PMNs, and macrophages will continue to produce inflammatory mediators in an effort to destroy the invaders. Unfortunately, these inflammatory mediators have a deleterious effect on the host tissue as well as foreign microbes. One of the effects of these mediators on the host is the induction of matrix degradation and bone resorption through activation of proteases and other inflammatory mediators that activate osteoclasts.

## 1. Introduction

Periodontal disease is an inflammatory process that affects almost 90% of the population and involves the supporting structure of teeth [1]. It is usually a progressively destructive change leading to loss of bone and periodontal ligament around the teeth which may eventually lead to their loss. The infection starts in the gingival epithelium leading to gingivitis, and under certain conditions it will progress into the underlying connective tissue leading to periodontitis [2]. Periodontal disease was found to be highly associated with other chronic inflammatory diseases such as cardiovascular disease, metabolic syndrome, diabetes, and rheumatoid arthritis increasing the risk of developing such diseases [3–6].

Until relatively recently, studying the pathogenesis of periodontitis focused on the role of bacterial infection. In the past two decades, there has been increasing interest in the host response as a factor that drives periodontal disease. It is now understood that the pathogenesis of periodontitis involves both innate and acquired immune response [7, 8]. The initial response to bacterial infection is a local inflammatory reaction that activates the innate immune system. The inflammatory response results in the release of an array of cytokines and other mediators and propagation of inflammation and recruitment of inflammatory cells into the gingival tissue. Spread of inflammation to the adjacent connective tissue drives the destruction of connective tissue and alveolar bone, that is, the cardinal sign of periodontal disease [9].

Although they may seem similar, the inflammatory and immune responses in periodontal tissue are quite different than those seen elsewhere in the body. For the most part, this is due to the unique anatomy of the periodontium and the unique character of the infection. The connective tissue of the periodontium is separated from the oral cavity by a thin permeable junctional epithelium that has remarkable cell and fluid dynamics that allow the flow of bacterial toxins and inflammatory mediators. Microbiome studies indicate that the defensive process in the periodontium occurs in response to a consortium of microbes that resides on the tooth surface in a biofilm community [10]. This contrasts with most other infections where the host contends with one organism [2, 11].

## 2. The Progressing Lesion of Gingival/Periodontal Tissue

Page and Schroeder divided the progression of gingival/periodontal lesion into four phases: initial, early, established, and advanced based on clinical and histological findings [12].

### 2.1. The Initial Lesion

The inflammation develops soon after plaque accumulation on the gingival third of the tooth. The initial lesion is a histological entity which would most likely correlate with a preclinical gingivitis. The initial lesion appears 2 to 4 days after plaque accumulation in previously healthy gingiva and is localized to the gingival sulcus, including the junctional epithelium and the most coronal part of the connective tissue. At this stage, there is marked dilation of the vasculature, elevated hydrostatic pressure in the microcirculation and increased gaps between endothelial cells in the capillaries which lead to increased permeability in microvascular bed. As a result, proteins and subsequently fluids start to exude into the tissue. Clinically, there is increased flow of gingival crevicular fluid (GCF) which helps in diluting and washing away noxious substances produced by the plaque biofilm. Also, during this initial phase, polymorphonuclear cells (PMNs) start to migrate into the area under the influence of adhesion molecules such as intercellular adhesion molecule-1 (ICAM-1) and endothelial adhesion molecule-1 (ELAM-1). They follow a chemoattractant gradient, formed by substances from plaque microflora and host cells, to the gingival crevice [11, 12].

### 2.2. The Early Lesion

After several days of plaque accumulation, the vessels in the dentogingival plexus increase in number. The increased size and number lead to the clinical sign of increased redness of the marginal gingival. Lymphocytes and PMNs are the predominant cells at this stage of gingivitis. Fibroblasts start to show signs of degeneration through apoptosis, and collagen fibers start to breakdown to provide space for leukocyte infiltration. The basal cell layer of the epithelium start to proliferate in an attempt to increase the physical barrier between the biofilm and the connective tissue. The epithelial rete pegs appear to be invading the underlying connective tissue [2, 11, 12]. This so-called early lesion may persist for long periods of time before progressing to an established lesion. This depends on many factors but mostly bacterial virulence and host susceptibility. Nevertheless, It has been shown that teeth with sites of persistent inflammation have 63.4% 50-year survival rate compared to 99.5% in teeth with no inflammation [13].

### 2.3. The Established Lesion

As the exposure to plaque bacteria continues, the inflammatory response in the gingival tissue continues to be enhanced. There is increased infiltration of leukocytes into the junctional epithelium and connective tissue. In young individuals, the predominant leukocyte in this infiltrate is lymphocytes, while in older subjects, the dominant cell type is plasma cells [14]. Collagen degradation continues while the inflammatory cell infiltrate expands deeper into the tissue. During this time, the dentogingival epithelium continues to proliferate and the rete pegs extend deeper into the connective tissue in an attempt to maintain epithelial integrity and a barrier to microbial entry. The junctional epithelium is replaced by loosely adherent pocket epithelium which allows for deeper migration of the bacterial biofilm. This pocket epithelium is heavily infiltrated with leukocytes, predominantly PMNs, and is more permeable allowing for passage of substances in and out of the connective tissue. This lesion may remain stable for months or years and may progress to a more destructive advanced lesion [2, 11, 12].

### 2.4. The Advanced Lesion

The inflammatory cell infiltrate, which is predominantly plasma cells, extends deeper into the connective tissue. This results in widespread manifestations of inflammation and immunopathological tissue damage. There is extensive degradation of collagen fibers and more apical migration of junctional epithelium. The pocket deepens and the biofilm continues to grow apically in an anaerobic environment. At this stage, loss of connective tissue attachment and bone loss occur [11].

## 3. The Immune Response and Periodontal Disease

The main purpose of the host response is to ward off invading pathogens. In some chronic diseases, such as periodontitis, this inflammatory response is part of the problem. The invading bacteria can cause periodontal tissue damage directly through proteinases and endotoxins and indirectly through provoking a host response. The inflammatory process occurring in periodontal disease is characterized by the infiltration of leukocytes, which limit the level of bacterial invasion and at the same time may be harmful to the tissue. There are a number of factors that promote leukocyte recruitment, including bacterial products, cytokines, cross-talk between innate and adaptive immune responses, chemokines, lipid mediators, and complement [15].

In addition to the leukocytes of the inflammatory infiltrate, the cells residing in the normal periodontium, including fibroblasts, junctional epithelial cells, and vascular endothelium (all of which function in the healthy periodontium to maintain homeostasis), can be hijacked by exposure to bacterial agents such as lipopolysaccharide (LPS), cytokines such as interleukin-1 (IL-1) or tumor necrosis factor-*α* (TNF-*α*), or prostaglandins such as prostaglandin E2 (PGE-2) and become major participants in tissue destruction [8, 16, 17].

It is hypothesized that the disease progression is due to a combination of several factors, including the presence of periodontopathic bacteria, high levels of proinflammatory cytokines, matrix metalloproteinases (MMPs), and PGE-2, and low levels of interleukin-10 (IL-10), transforming growth factor-*β* (TGF-*β*), and tissue inhibitors of metalloproteinases (TIMPs). In this concept, it is clear that the balance of cytokines determines whether tissue destruction occurs or homeostasis is maintained [18].

The role played by bacterial pathogens in determining the progression of the disease and periodontal breakdown is highly complex. For one thing, the disease is not associated with a single species, but rather it involves a wide range of pathogenic bactria. Studying the oral microbiome revealed that periodontal disease is consistently associated with higher proportions of anaerobic and Gram-negative bacteria, such as *Prevotella*, *Leptotrichia, Veillonella*, *Porphyromonas*, and *Treponema* than periodontal health [19]. Such bacteria can cause tissue destruction directly through pathogenic products such endotoxins and collagenases or by provoking an immune response.

The first conclusive evidence that the host response played an important role in periodontal breakdown was demonstrated on beagles by using a potent cyclooxygenase inhibitor (flurbiprofen) which reduced the amount of bone loss caused by periodontal disease [20]. In other reports, inhibition of IL-1 and TNF-*α* reduced the recruitment of inflammatory cells (notably monocytes and lymphocytes) toward the periodontium [21]. As a result, there was a decrease in periodontal attachment loss and bone resorption [22, 23]. In later years, various knock-out mouse models supported the hypothesis that cytokines are integral in the disease process.

One of the critical components of the host response is a family of receptors called the toll-like receptors (TLRs) which detect the presence of bacteria. Activation of the innate immune response by the binding of various bacterial components (i.e., diacyl lipopeptides, peptidoglycan, LPS, flagellin, and bacterial DNA) to TLRs results in the production of cytokines and chemokines. Once TLRs are activated, an intracellular signaling cascade is stimulated that leads to the activation of transcription factors such as nuclear factor-*κ*B (NF-*κ*B), activator protein-1 (AP-1), and p38 and the production of various cytokines, many of which directly or indirectly stimulate osteoclast formation [24].

## 4. Immune Cells and Periodontal Disease

### 4.1. Lymphocytes

Lymphocytes are important immune cells that can produce IL-1, -6, and -17; receptor activator of NF-*κ*B ligand (RANKL); and TNF-*α* cytokines. As mentioned previously, it is one of the most prominent leukocytes in the inflammatory infiltrate associated with periodontal degradation [25]. lymphocytes also secrete a number of inhibitory molecules that directly inhibit osteoclast formation, including osteoprotegerin (OPG); IL-4, -10, and -13; and interferon-*γ* (IFN-*γ*) [25]. It is now generally agreed that both T and B cells are present in periodontal disease tissues, and both T and B cells extracted from gingival tissues have been reported to be at a more advanced stage of the cell cycle than peripheral blood T and B cells, indicative of activation within the tissues [18].

There is evidence to suggest that lymphocytes are involved in enhancing periodontal disease induced bone loss. When severe combined immunodeficient mice that lack B and T lymphocytes were challenged with *P. gingivalis*, they exhibited considerably less bone loss than immunocompetent mice. This result suggested that B and T lymphocytes are not critical for protecting the host against *P. gingivalis* but do contribute to bone loss when present [26].

In an attempt to identify the cellular source of RANKL in the bone resorptive lesions of periodontal disease, Kawai et al. measured the concentrations of soluble RANKL in diseased tissue homogenates. RANKL concentrations were significantly higher in diseased gingival tissues compared to healthy tissues. Double-color confocal microscopic analyses demonstrated less than 20% of both B cells and T cells expressing RANKL in healthy gingival tissues. By contrast, in diseased gingival tissues, more than 50 and 90% of T cells and B cells, respectively, expressed RANKL. Moreover, lymphocytes isolated from gingival tissues of patients induced differentiation of mature osteoclast cells in a RANKL-dependent manner in vitro [27]. This indicates that activated T and B cells can be the cellular source of RANKL for bone resorption in periodontal diseased gingival tissue.

Activated B cells have been shown to contribute to periodontal bone destruction. Han et al. evaluated the effect of *A. actinomycetemcomitans*-responsive B lymphocytes on periodontal bone resorption and their level of RANKL expression. They demonstrated that B cells from *A. actinomycetemcomitans*-immunized animals had greater levels of RANKL expression and induced a significantly higher level of osteoclast differentiation than nonimmune B cells that were not Ag specific. They also show that these animals exhibited increased osteoclast formation on the alveolar bone surface and significant periodontal bone resorption. This effect was antagonized by injection of osteoprotegerin fusion protein into the local gingival tissues. This study suggests that B lymphocytes can contribute to increased periodontal bone resorption and that this effect is associated with the upregulation of RANKL expression [28].

One of the pathogenic pathways being suggested is the immune responses to self-antigens such as collagen type I, a major component of the periodontium. This is suggested because periodontitis patients have higher numbers of anticollagen type I antibody producing cells in the gingiva compared to peripheral blood [29]. Furthermore, anticollagen type I specific T-cell clones were identified in the inflamed gingival tissues of periodontitis patients [30]. It was also demonstrated that there was an increase in the number of autoreactive CD5+ B cells in periodontitis lesions, and the CD5+ B cells produced more IgM and IgG antibodies to collagen in vitrothan did CD5− B cells [31]. Although the mechanisms that induce an immune response to self-components are not fully elucidated, molecular mimicry has been suggested to explain the linkage between infection with bacteria and subsequent autoimmune mechanisms [32].

During the chronic phase of the disease, the lymphocytic response has been suggested to be generally protective, to facilitate bacterial clearance, and to arrest disease progression. Studies have shown that antibody depletion of B lymphocytes in normal rats increased alveolar bone loss in an *Actinomyces viscosus* and *Bacteroides gingivalis* gavage model [33]. In humans, serum from patients with severe periodontitis containing high titers of anti-*P. gingivalis* antibodies completely inhibited in vitro bone resorption, whereas serum from patients with low titers failed to inhibit this bone resorption, confirming a possible protective role for specific antibodies in periodontitis [34]. On the other hand, antibody levels to subgingival plaque microorganisms were positively correlated to periodontal bone loss and were found to be a predictor of bone loss in an elderly group of patients [35]. These results indicate that specific antibodies produced in response to periodontopathic bacteria are protective. However, susceptible subjects are characterized by the production of nonprotective antibodies.

CD4+ T cells are important in determining the effect of the T-cell immune responses against pathogens. Effector CD4+ T cells are classified into Th1 and Th2 subsets. Cytokines produced by Th1 lymphocytes include IFN-*γ* and TNF-*α* and -*β*. They are critical for the eradication of intracellular pathogens and are generally proresorptive through direct or indirect effects, whereas Th2 cytokines, such as IL-4 and IL-10, are not. The adoptive transfer of antigen-specific Th1 cells resulted in the enhancement of periodontal bone loss in rats and mice stimulated by bacteria [36, 37]. In contrast, recipient rats that received the adoptive transfer of Th2 cells exhibited less bone loss [38]. These studies suggest that Th2 cells could be protective, providing help for specific antibody production which is a key feature of protection against periodontal destruction.

Based on the fact that there is a shift in lymphocyte populations in the inflammatory infiltrate from predominant T cells in gingivitis to an increased proportion of B cells in periodontitis, some authors suggest that the susceptibility to periodontal disease progression may involve predominantly Th2 cells which produce the cytokines required for B-cell proliferation and differentiation, leading to polyclonal B-cell activation, the production of elevated levels of nonprotective antibodies, and the continued production of B-cell IL-1. Nonsusceptibility to periodontal breakdown may involve predominantly Th1 which is involved in T-cell activation, cell mediated immunity, IFN-*γ* enhancement of innate immunity, and, if necessary, the production of protective antibodies [39].

Another subset of effector T helper cells that has recently been implicated in the pathogenesis of periodontal disease is T helper type 17 cells (Th17) which are distinguished by the production of interleukin-17 (IL-17). Immunohistochemical analysis revealed increased presence of these cells in periodontitis diseased tissue compared to gingivitis or periodontally healthy tissue [40, 41]. The presence of these cells was associated with increased expression of IL-17, IL-6, and RANKL [41]. IL-17 is crucial in the protection against extracellular pathogens and may play a role in promoting alveolar resorption when released in excessive amounts. IL-17 exerts its osteoclastogenic activity by enhancing RANKL expression on osteoblasts and CD4+ T cells [42]. Furthermore, IL-17 contributes to local inflammation by recruiting and activating immune cells, leading to an abundance of inflammatory cytokines, such as IL-1, and TNF-*α*, and RANKL [43].

Overall, these data suggested that the adaptive immune response and, in particular, CD4+ T cells and the proinflammatory cytokines that they secrete are important effectors of bone loss as a result of bacterial infection. Although many cross-sectional studies have explored the production of Th1 and Th2 cytokines in human periodontal disease, few longitudinal studies linked the expression of these cytokines with the loss of periodontal bone or attachment. 

### 4.2. Neutrophils

Neutrophils or PMNs have been described as playing a major role in periodontal disease. They have been shown to have both protective and destructive influences [44]. Evidence that propose a protective function is based on the observation that individuals who have neutrophil disorders such as cyclic neutropenia [45], Chédiak-Higashi syndrome [46], and leukocyte adhesion deficiency syndrome [46] have an increased susceptibility to periodontal destruction. Further, MacFarlane et al. reported significantly impaired phagocytosis due to a decreased rate of adhesion and opsonization by neutrophils from patients with refractory periodontitis compared with healthy patients [47]. In the sulcus, neutrophils form a barrier between the epithelium and plaque which may prevent bacterial invasion of the epithelium and underlying connective tissue [48]. 

Relatively recently, a new concept emerged in which PMN hyperactivity, rather than hypoactivity, and excess release of toxic products by these cells are at least partly responsible for periodontal tissue destruction. The respiratory burst is an important pathway for microbial killing and involves the generation of superoxide, hydrogen peroxide, hydroxyl radical and, subsequently, hypochlorous acid, and chloramines. These enzymes are responsible for oxidative killing inside the phagosome and may be released into the extracellular microenvironment increasing the oxidative stress in the vicinity [49]. Oxidative stress manifested along with elevated cytokine levels in close proximity to alveolar bone can activate forkhead box O3 and Wnt signaling pathways, which in turn trigger RANKL-mediated bone resorption [50].

Extracellular killing contributes to the elimination of the invading microorganism but at the same time can also damage the adjacent cells and tissues. One way of extracellular killing that has been of interest in recent years is called neutrophil extracellular traps. In that process, the neutrophil undergoes a sequence of events that leads to the release of its entire nuclear chromatin (DNA and associated histone-rich protein backbone). After being mixed with granular cathelicidin antimicrobial peptides, it is actively extruded by the neutrophil as a type of biologic “spiders web” into the extracellular space or tissue. These traps act by trapping and immobilizing pathogens preventing tissue and systemic spread. Pathogen killing is accomplished by neutrophil extracellular trap-embedded cathelicidin antimicrobial peptides. The cytotoxic effect of neutrophil extracellular traps is not limited to foreign pathogens as the host's own endothelial and epithelial cells have been shown to be susceptible to neutrophil extracellular traps and their DNase-generated neutrophil extracellular trap fragments [49]. 

PMN degranulation also results in the release of elastases and collagenases which hydrolyze several extracellular proteins such as elastin, fibronoctin, and collagen. Experiments by Lee et al. using specific enzyme inhibitors to identify the pattern of collagen substrate degradation demonstrated that the collagenase activity in progressive periodontitis was derived from neutrophils and not from bacteria or other host cells. In addition, active collagenase activity was higher in patients with progressive loss of connective tissue compared to the groups with inflamed tissues alone. Moreover, the ratio of active to total collagenase activity was 50% higher in the group with progressive lesions [51]. Cleavage of matrix molecules by collagenases and elastases may generate peptide fragments that are chemotactic for monocytes. In addition, PMNs have the potential to release proinflammatory mediators that have numerous biological activities [52].

### 4.3. Macrophages/Monocytes

Macrophages are important mediators of inflammation in the connective tissue infiltrate where they produce several cytokines and also present antigens to T cells [44]. They are an essential part of the innate immune response to intracellular infection. They produce proinflammatory cytokines which enhance phagocytosis and in most cases result in the successful elimination of the pathogen [53]. In addition, macrophages/monocytes are capable of differentiating to osteoclasts in response to TNF-*α* in the presence of RANKL. This means that these cells form a key link between the immune system and bone resorption [54]. 

In periodontal disease, macrophages/monocytes are major contributors to tissue breakdown. Samples from periodontitis patients had higher numbers of macrophages/monocytes associated with greater collagen breakdown and higher level of MMPs compared to controls [55]. Studies have shown that IL-1 was expressed predominantly by macrophages in tissue isolated from periodontal patients [56]. Using an immunohistochemistry technique, Crotti et al. demonstrated that significantly higher levels of RANKL protein are associated with macrophages in the periodontitis tissues [57]. Periodontal pathogens, such as *A. actinomycetemcomitans* and *P. gingivalis*, have been shown to activate monocytes and macrophages and stimulate the secretion of proinflammatory and tissue-destructive mediators such IL-1, TNF-*α*, IL-6, and PGE2 [16, 58, 59]. These studies demonstrate that one of the responses of macrophages to bacterial invasion of periodontal tissue is production of inflammatory mediators that contribute to the destruction of tissue components including bone.

## 5. Inflammatory Cytokines and Periodontal Disease

Offenbacher in 1996 suggested that if the antibody/neutrophil response does not result in clearance, the outcome of the monocyte/lymphocyte challenge is the secretion of catabolic cytokines and inflammatory mediators such as IL-1, IL-6, TNF-*α*, and PGE2, which induce connective tissue and bone loss [60].

### 5.1. IL-1

IL-1 is a proinflammatory cytokine that has a large array of activities that regulate the inflammatory process [61]. There are two forms of IL-1 that have agonist activity, IL-1*α* and IL-1*β*. For control purposes there is a third ligand called IL-1 receptor antagonist (IL-1RA) that functions as a competitive inhibitor. There are surface receptors on the surface of target cells, designated IL-1 receptor-1 (IL-1R-1) and IL-1 receptor-2 (IL-1R-2) [61]. IL-1R-1 is thought to mediate most of the functions of IL-1 and IL-1R-2 functions mostly as a decoy receptor [62]. IL-1R-2 can also be cleaved from the cell surface at the site of inflammation to function as an indigenous inhibitor of IL-1 [63]. In the periodontium, IL-1 was found to be produced by several types of cells such as PMNs, monocytes, and macrophages [64–66]. 

The role for IL-1 in mediating bone loss stimulated by periodontal pathogens has been comprehensively studied in human and animal models. In patients with periodontal disease, IL-1*β* expression was elevated in the GCF at sites of recent bone and attachment loss [17, 67, 68]. IL-1*β* was also found to be higher in diseased periodontal tissue samples compared to tissue samples from healthy individuals, and the level of expression correlates highly with clinical parameters [64, 69]. Injecting bacterial LPS into the gingiva of an animal model induced increased inflammatory infiltrate, significantly increased immunostaining for IL-1*β*, and more TRAP-positive osteoclasts and led to increased bone loss [70].

Investigators have shown that inhibiting IL-1 causes a decrease in bone loss, while increasing IL-1 extenuate bone resorption. Using a nonhuman primate model, Delima et al. showed that inhibition of IL-1 using human soluble IL-1 receptor type I significantly reduced inflammation, connective tissue attachment loss, and bone resorption induced by periodontal pathogens compared to controls [71]. In other studies, IL-1 receptor deficient mice had less *P. gingivalis* LPS-induced osteoclastogenesis compared to similarly treated wild-type mice [72]. In a different approach, the exogenous application of recombinant human IL-1*β* in a rat ligature induced periodontitis model accelerated alveolar bone destruction and inflammation over a two-week period [73]. In addition, transgenic mice overexpressing IL-1*α* in gingival epithelium developed a periodontitis-like syndrome, leading to loss of attachment and destruction of alveolar bone [74]. 

IL-1 expression is correlated to the expression proreserbative cytokines and proteinases. The expression of MMP-1, -2 and -9 and RANKL was correlated with the expression of IL-1*β* in a time period characterized by the intense increase of inflammatory reaction and alveolar bone loss [75]. Osteoclast formation, expression of RANK, EP4, and Cox2 mRNAs and production of PGE2 were significantly increased in IL-1R antagonist (IL-1Ra) knockout mice stimulated with *A. actinomycetemcomitans* LPS compared with wild-type mice [76]. Taken together, these studies strongly support the role of IL-1 in promoting alveolar bone destruction in periodontal disease. 

There is evidence that susceptibility to periodontal disease is influenced by genetic polymorphism of the IL-1 gene. These studies report on an association between IL-1 composite genotype and the severity of periodontal disease [77]. Although other studies failed to demonstrate such an association [78]. A recent meta-analysis demonstrated that IL-1A and IL-1B genetic variations are significant contributors to chronic periodontitis [79]. Nevertheless, large cohort studies of homogenous composition are needed.

### 5.2. TNF-*α*

TNF refers to two associated proteins, TNF-*α* and TNF-*β*. There are two structurally similar TNF cell surface receptors, TNF receptor-1 (TNFR-1) and TNF receptor-2 (TNFR-2) [80]. These receptors activate different signaling pathways and have different cytoplasmic domains. Most of the inflammatory effect is mediated through TNFR-1 signaling, while TNFR-2 attenuates the inflammatory response induced by TNF [81]. 

TNF-*α* plays an important role in periodontal disease as demonstrated by human studies that show an increased expression TNF-*α* during periodontal breakdown. In patients with periodontal disease, TNF-*α* expression was elevated in the GCF at sites where bone and attachment loss have just occurred [17, 67, 68, 82, 83]. It was also found to be higher in diseased periodontal tissue samples compared to tissue samples from healthy individuals [69]. In rats, *A. actinomycetemcomitans* LPS gingival injections induced severe bone loss, increased inflammatory infiltrate, significantly increased immunostaining for TNF-*α*, and more TRAP-positive osteoclasts [70]. Hence, TNF-*α* is present during periodontal disease-associated bone resorption.

A cause and effect relationship can be established between TNF-*α* and periodontal bone loss by increasing TNF-*α* levels using recombinant technology and inhibiting TNF-*α* or its receptor. For instance, the administration of recombinant TNF-*α* caused acceleration in the progression of periodontal destruction in a rat ligature induced periodontitis model [84]. On the other hand, *P. gingivalis*-induced osteoclastogenesis was reduced in TNF receptor-deficient mice compared to wild-type controls, indicating that osteoclast formation is dependent on a TNF-*α* regulated pathway as a part of the host response to bacterial challenge [85]. In a study by Garlet et al., TNFR-1 knockout mice developed significantly less inflammation, indicated by decreased expression of chemokine and chemokine receptors, and less alveolar bone loss in association with decreased expression of RANKL in response to *A. Actinomycetemcomitans* oral inoculation. The apparent level of *A. actinomycetemcomitans* quantified by real-time PCR was significantly greater in the TNF receptor ablated mice compared to wild-type controls [86]. Decreased inflammatory response and bone destruction were also observed when TNF-*α* was inhibited using a soluble antagonist [22]. Taken together, the decrease in TNF-*α* seemed to reduce the host response, thereby leading to higher levels of bacteria, but at the same time there was less net bone loss. In contrast, when exogenous TNF was added there was more periodontal bone loss indicating that the level of TNF-*α* is directly related to the amount of tissue destruction.

To investigate the mechanisms involved in TNF-*α* induced bone loss, studies examined the correlation between TNF-*α* and other preservative mediators. In the aforementioned study, TNFR-1 deficient mice had lower levels of the neutrophilic antimicrobial myeloperoxidase in response to *A. actinomycetemcomitans* infection. Furthermore, the quantitative analysis of mRNA expression from inflammatory cytokines IL-1*β*, IFN-*γ*, and RANKL in gingival tissues revealed that it was significantly lower in infected TNFR-1 knock-out mice compared to wild-type infected mice. Thus, the absence of TNFR-1 resulted in a lower production of cytokines in response to *A. actinomycetemcomitans* infection [86]. In another study by the same group, oral inoculation of *A. actinomycetemcomitans* resulted in an intense and widespread migration of leukocytes to the gingival tissues along with marked alveolar bone resorption. The expression of MMP-1, -2, and -9 and RANKL was correlated with the expression of TNF-*α* during an intense inflammatory reaction and alveolar bone loss [75]. In vitro studies show that TNF-*α* caused an increased expression of MMP-1, -3, and -13 and cyclooxygenase-2 (COX-2) [87]. Based on studies like these, it can be projected that TNF-*α* plays a role in controlling the expression of the cytokines that stimulate bone resorption during periodontal disease. 

### 5.3. IL-6

In the gingiva, IL-6 is produced by mononuclear cells. These cells showed higher expression of IL-6 in response to periodontal pathogens compared to their peripheral blood counterparts [88]. Macrophages were shown to secrete IL-6 in response to *P. gingivalis* LPS in a dose-dependent manner [16]. In addition, T cells extracted from periodontitis patient expressed more IL-6 compared to T-cell from healthy controls [89]. IL-6 is also one of the cytokines found in the GCF of patients with refractory periodontitis who are undergoing active bone loss [90].

The association between IL-6 and alveolar bone loss in periodontal disease was also established in animal models. *A. actinomycetemcomitans* LPS induced severe bone loss associated with increased inflammatory infiltrate, increased immunostaining for IL-6, and more TRAP-positive osteoclasts [70]. In response to *P. gingivalis* oral inoculation, mice with genetically deleted IL-6 had decreased bone loss compared to wild-type mice [91]. These studies indicate that the production of IL-6, which is proinflammatory, contributed to periodontitis induced bone resorption. 

### 5.4. INF-*γ*

INF-*γ* is a lymphokine produced by activated T lymphocytes and natural killer cells that play an important role in host defense mechanisms by exerting pleiotropic activities on a wide range of cell types. Cellular responses to INF-*γ* are mediated by its heterodimeric cell surface receptor (IFN-*γ*R), which activates downstream signal transduction cascades, ultimately leading to the regulation of gene expression. 

As an inflammatory cytokine, INF-*γ* was studied as mediator of periodontal destruction in animal and human studies. INF-*γ* knock-out mice showed decreased bone loss in response to *P. gingivalis* infection compared to wild-type controls [91]. In addition, T cells extracted from periodontitis patient expressed more INF-*γ* compared to T cell from healthy controls [89]. In an animal study, IFN-*γ* was associated with enhanced alveolar bone loss mediated by RANKL-expressing CD4(+) Th cell in response to *A. actinomycetemcomitans* during the progression of periodontal disease and a concomitant and significantly increased coexpression of IFN-*γ* in RANKL(+) CD4(+) Th cells [92].

### 5.5. RANKL/OPG

RANK, RANKL, and OPG are cytokines that belong to TNF-*α* super family. RANK is a receptor found on the surface of osteoclast precursors. When RANK binds to its ligand RANKL, it stimulates the differentiation of these precursor cells into mature osteoclasts. OPG competes with RANKL by binding to RANK without stimulating any differentiation. It is the ratio of RANKL and OPG expressions that is important in inflammation induced bone resorption, including periodontitis [93].

How the relative concentrations of RANKL and OPG are altered during the progression periodontal disease was investigated in mice orally inoculated with *A. actinomycetemcomitans*. Infiltration of leukocytes within periodontal connective tissue and increase in leukocyte count were observed followed by a rapid increase in alveolar bone loss. An increase in the concentrations of inflammatory cytokines, MMPs and RANKL, was observed during this time. However, during later points, in which a slower rate of bone loss was observed, the concentrations of proinflammatory cytokines, MMPs, and RANKL decreased. Instead, there was a dramatic increase in the concentrations of anti-inflammatory cytokines (e.g., IL-4 and -10), as well as TIMPs and OPG. Thus, the bone loss observed correlated with an expression pattern in which RANKL was increased relative to OPG over the early part of the study, while during the latter part of the study in which the rate of bone loss slowed, there was a marked decrease in RANKL concentration, whereas OPG concentration was at its highest [75].

A number of clinical studies investigated the concentrations of RANKL and OPG in gingival tissues or GCF extracted from individuals with periodontitis to determine the RANKL/OPG ratio. These studies show an increase in soluble RANKL, with or without a decrease in OPG, with chronic periodontitis compared to healthy controls [27, 57, 94]. The trend was generally that RANKL/OPG ratio was higher in individuals with periodontitis than in healthy controls. In a rodent ligature induced periodontitis model, treatment with OPG reduced periodontal bone loss following adoptive transfer of lymphocytes and infection with *A. actinomycetemcomitans *[95]. These findings correspond well with the critical role of RANKL in driving osteoclastogenesis and bone loss in periodontal disease.

### 5.6. IL-17

Although Th17 express RANKL, they may not activate osteoclasts by a RANKL-RANK interaction. Rather, secretion of IL-17 by Th17 cells and induction of RANKL on cells such as osteoblasts that support the activation of osteoclasts appear to be required for bone loss. Through the RANKL-RANK system, IL-17 may have a role in rheumatoid arthritis, periodontal disease, and loosening of joint prostheses [96]. However, a study examined the role of IL-17 in inflammatory bone loss induced by the oral pathogen *P. gingivalis* in IL-17 receptor-deficient mice. These mice showed enhanced periodontal bone destruction, suggesting a bone-protective role for IL-17 [97].

Conversely, some evidence implicates IL-17 in the destructive phase of periodontal disease. In a clinical study, IL-17 expression was significantly higher in periodontal lesions, especially in the tissue adjacent to bone destruction, than in control sites [98]. IL-17 regulates MMPs and inflammatory cytokines in gingival fibroblasts, and *P. gingivalis* can stimulate IL-17 production from T cells in vitro [99, 100]. IL-17 also regulates COX-2 and PGE2 production [101, 102]. Another possible destructive role for Th17 cells is supported by recent findings that implicate Th17, rather than Th1, as the specialized osteoclastogenic lymphocyte that links T-cell activation to bone resorption [103]. Ultimately, studies of periodontitis in humans treated with anti-IL-17 biologics may provide the final answer as to the role of the IL-17 in human periodontal disease.

## 6. Chemokines and Periodontal Disease

Chemokines are a large family of chemotactic cytokines that stimulate the recruitment of inflammatory cells. They are divided into two major families based on their structure, CC and CXC chemokines. They act through receptors referred to as CC chemokine receptor (CCR) and CXC chemokine receptor (CXCR). Chemokines are produced by several cell types in the periodontium, such as fibroblasts, endothelial cells, macrophages, osteoclasts, epithelial cells, polymorphonuclear leukocytes, monocytes, lymphocytes, and mast cells. Some chemokines can stimulate one or more steps of bone resorption, including the recruitment, differentiation, or fusion of precursor cells to form osteoclasts or enhance osteoclast survival [104]. They could also affect periodontal bone loss by recruiting cells, such as neutrophils, which protect against bacterial invasion [9, 105]. 

Chemokines were found in both gingival tissue and crevicular fluid during the immunopathogenesis of periodontal diseases. IL-8/CXCL8, a chemoattractant of PMNs, showed a rapid increase in GCF preceding the clinical signs of periodontal disease following cessation of tooth brushing [106]. In persons with periodontitis, the levels of IL-8/CXCL8 in both periodontal tissue and GCF are drastically increased and have been correlated with disease severity [107]. In vitro studies have demonstrated that IL-8/CXCL8 can be produced by gingival fibroblasts, gingival epithelial cells, and endothelial cells [105]. Another chemokine that could contribute to the enhanced severity of periodontal disease is macrophage chemoattractant protein-1 (MCP-1/CCL2), which is supposed to be the major chemoattractant of macrophages in periodontal diseases. This is supported by analysis of data showing that MCP-1/CCL2 activity in GCF increased with severity of the disease [108].

The chemokine called (regulated upon activation, normally T-cell expressed and secreted) (RANTES/CCL5) has also been detected in both the periodontal tissue and the GCF of persons with periodontitis, and in higher amounts in active sites compared to inactive periodontitis sites [109, 110]. Cell cultures of whole blood from persons with periodontitis produce higher levels of RANTES/CCL5 when stimulated with LPS compared to cultures from control individuals. Even after therapy, persons with periodontitis were found to continue producing high levels of RANTES/CCL5 [111]. This indicates that these individuals have intrinsic susceptibility to periodontitis development. One of the most abundantly expressed chemokine in periodontitis tissues is macrophage inflammatory protein-1*α* (MIP-1*α*/CCL3) with its expression localized in the connective tissue subjacent to the pocket epithelium of inflamed gingival tissues [110]. It has also been shown that MIP-1*α*/CCL3-positive cells increase in number with increasing severity of periodontal disease [110, 112]. The receptor of RANTES/CCL5 and MIP-1*α*/CCL3, CCR5, was found to be exclusively expressed in diseased tissues, mainly in cells located in connective tissue subjacent to the pocket epithelium [113]. 

As previously discussed, chemokines can also exert important effects on bone cells, inducing the migration and activation of osteoclasts. MIP-1*α*/CCL3, described as an osteoclast differentiation factor, and RANTES/CCL5, a chemotactic factor for these cells, are found in periodontitis tissues [104, 114–116]. In addition, stromal-cell derived factor-1 (SDF1/CXCL12) has also been described as a positive regulator of osteoclast function and was recently identified in diseased periodontium [117]. Therefore, the presence of these osteoclast chemoattractants in the periodontal environment may be involved in the exacerbation of disease severity and bone loss.

## 7. Prostaglandins and Periodontal Disease

The cyclooxygenase enzymes COX-1 and COX-2 catalyze the conversion of arachidonic acid to prostaglandins. COX-1 is a constitutive enzyme responsible for the formation prostaglandins with physiological functions, while COX-2 is an inductive enzyme induced primarily by proinflammatory cytokines and leads to the formation of prostaglandins involved in pathophysiological processes such as edema formation and fever. Prostaglandins are potent stimulators of bone formation and resorption and are produced by osteoblasts and PDL cells. Prostaglandins also have inhibitory effects on fully differentiated osteoblasts and osteoclasts [118, 119]. 

The role of prostaglandins in the pathogenesis of periodontitis has been under investigation in recent years. PGE-2 and leukotriene B-4 were found in GCF of localized juvenile periodontitis (now called localized aggressive periodontitis), suggesting a role of these molecules in periodontal disease. Furthermore, when *P. gingivalis* was introduced within murine dorsal air pouches, it elicited leukocyte infiltration, concomitant with elevated PGE-2 levels in the cellular exudates and increased COX-2 expression in infiltrated leukocytes [120]. Recently, macrophages were shown to secret more PGE2 when stimulated with *P. gingivalis* LPS in a dose-dependent manner [16]. 

In an animal ligature induced periodontitis model, both a nonselective COX inhibitor and a selective COX-2 inhibitor reduced osteoclast numbers and alveolar bone loss compared to nontreatment [121]. Many clinical trials explored the use of a COX-2 inhibitor as an adjunct to periodontal therapy. These inhibitors improved the clinical outcome after periodontal therapy compared to periodontal therapy alone [122, 123]. These studies indicate that prostaglandins, especially PGE-2, may be involved in periodontitis induced bone resorption.
